# The future of sleep apnea management: we can either ride the bus or drive it

**DOI:** 10.3389/frsle.2023.1323447

**Published:** 2024-01-12

**Authors:** Stephen D. Pittman, Barry Chase, Daniel J. Gottlieb, Dennis Hwang, Douglas B. Kirsch, Neomi A. Shah, Kimberly L. Sterling, Keith Thornton, Teresa R. Barnes, John Tosi, Kelly A. Carden, Richard K. Bogan, Amir Reuveny, Sonia Ancoli-Israel, Atul Malhotra

**Affiliations:** ^1^Apnimed, Cambridge, MA, United States; ^2^Chase Dental Sleepcare, Commack, NY, United States; ^3^VA Boston Healthcare System and Brigham and Women's Hospital, Boston, MA, United States; ^4^Kaiser Permanente, Fontana, CA, United States; ^5^Sleep Medicine Atrium Health and Department of Neurology, Wake Forest University School of Medicine, Charlotte, NC, United States; ^6^Division of Pulmonary, Critical Care, and Sleep Medicine, Department of Medicine, Icahn School of Medicine at Mount Sinai, New York, NY, United States; ^7^ResMed Science Center, San Diego, CA, United States; ^8^SleepWell Solutions, Dallas, TX, United States; ^9^Respiratory Research Advocate and Patient Advocate, St. Louis, MO, United States; ^10^John Tosi DDS, Private Practitioner, St. Louis, MO, United States; ^11^Ascension Medical Group, Nashville, TN, United States; ^12^Bogan Sleep Consultants, Columbia, SC, United States; ^13^Wesper, New York, NY, United States; ^14^Department of Psychiatry, University of California San Diego, La Jolla, CA, United States; ^15^Division of Pulmonary, Critical Care, Sleep Medicine, and Physiology, Department of Medicine, University of California San Diego, La Jolla, CA, United States

**Keywords:** obstructive sleep apnea (OSA), continuous positive air pressure (CPAP), home sleep apnea test (HSAT), value-based care, patient journey, oral appliance therapy

## Abstract

This consensus conference report summarizes discussions on sleep apnea care and management. Our goal is to simplify the journey to optimize success for individuals at risk of obstructive sleep apnea and to facilitate diagnostics, monitoring and communication among the entire healthcare team including patients, primary care physicians, sleep specialists, sleep dentists and other key providers. The statement identifies five key problems or unmet needs and contemplates four potential future directions.

Our goal is to simplify the journey to successful diagnosis and treatment of obstructive sleep apnea (OSA) and to facilitate communication among providers and between patients and providers. Such providers may include primary care physicians, sleep specialists, sleep dentists and others.

OSA is thought to affect up to 1 billion people worldwide although, due to logistics, current diagnostic strategies are challenging (Benjafield et al., 2019). The current gold standard approach of seeing a board-certified sleep specialist followed by polysomnography or home sleep apnea testing with a technically adequate device is unlikely to be scalable to assess the global burden of disease particularly given that many more patients are at risk of OSA. Further complicating scalability is the expected increase in prevalence due to the obesity pandemic and the aging of the population (Mulgrew et al., 2007; Rosen et al., 2012). We convened a consensus conference of key stakeholders to discuss potential future approaches to sleep apnea care and management. The conference was sponsored by Wesper, but the sponsor had no role in guiding the discussion or summarizing conclusions which were at the discretion of the co-chairs (SDP, AM).

## 1 Introduction

The group met via Zoom teleconference in March 2022 with a robust discussion moderated by the co-chairs. Three session topics were used to guide discussions: personnel + communication, equipment, and financial models. Documents were circulated to seek input yielding this proceedings summary. The participants were chosen to provide diversity both from the perspective of training background (e.g., dentist, physician, technologist, health economist, and patient advocate) and various health systems (e.g., Veteran Affairs, Kaiser Permanente, academic medical centers, non-academic medical centers, private practice, and industry). We further sought input via follow-up calls from key opinion leaders who were unable to participate in the interactive discussion, but whose opinions were valued. Their comments contributed to the final summary. A few topics like financial models were taken offline to dive deeper into these topics with subject matter experts. The details of these more granular topics were summarized in this final document.

## 2 Problems and unmet needs

The current framework for this document uses the conference discussions to define problem(s) and identify unmet needs rather than attention on specific solutions. Our goal in agreeing on problems and unmet needs was to establish a strong foundation or baseline on which to propose future directions toward solutions for the benefit of all OSA patients.

### 2.1 Patient journey needs to be simplified

At present, some health systems encourage an initial evaluation by a primary care physician followed by a sleep consultation and evaluation with a specialist (Hwang et al., 2018). The specialist may then order a polysomnogram or home sleep apnea test. The patient is scheduled for a subsequent follow-up visit where the patient returns to the specialist to discuss the results and may be prescribed treatment, usually PAP therapy. Though some practices incorporate the distribution of durable medical equipment (DME) into their clinical flow, oftentimes the patient receives the PAP therapy through a home care company, only to have to return to the specialist a few months later for monitoring and evaluation. While multiple visits may be appropriate, they can be perceived as burdensome to patients and thus alternative care models to improve efficiency may be well received. In principle, a subset of patients at risk of OSA could undergo diagnostic testing at the discretion of a provider or at the request of a concerned patient. In such a model, patients could be prescribed therapy without the need for multiple visits to confirm what may be clinically obvious. One common example is the high volume of patients undergoing bariatric surgery (Lee et al., 2009; Sareli et al., 2011; Rodbard, 2016; Horvath et al., 2018; Kreitinger et al., 2020; Raphelson et al., 2022). Such patients have high pre-test probability for OSA and the data suggest increased risk of perioperative complications in people with OSA undergoing bariatric surgery (Glazer et al., 2018; Ahlin et al., 2019). Thus, a reasonable approach could involve an initial diagnostic test in these patients without the need for additional visits for routine cases. One challenge exists when serial nights of data are required for optimal patient care (Lechat et al., 2023). Currently, the patient would repeatedly return to clinic or receive serial mailings to get diagnostic equipment because most existing medical technology does not allow serial assessments over multiple nights. While objective testing is an important aspect of the patient journey, explanation of testing results, review of appropriate treatment options, and long-term monitoring of therapeutic outcomes remain essential steps in the journey to ensure optimal patient outcomes.

### 2.2 Enhanced communication is needed

A number of participants expressed concern about the lack of communication that sometimes occurs between various providers e.g., dentists and doctors, sleep providers and surgeons, providers and durable medical equipment (DME) companies, etc. In some cases, the participants described competition rather than collaboration despite general acknowledgment that the pool of patients potentially seeking treatment is vast. Secure communication is often a challenge since various providers may not use the same electronic health records system, e.g., many dental offices use dental software that does not typically integrate with medical electronic health record (EHR) platforms. Alternative communication methods such as text messaging and email may not be HIPAA compliant. In some cases, experienced providers reported receiving the most difficult cases (sometimes described using the pejorative term “train wrecks”) after other therapies had been exhausted. For example, patients who are morbidly obese and have failed positive airway pressure (PAP) therapy going to a dentist for an oral appliance as a last resort (Mohammadieh et al., 2022). There was general agreement that secure communication strategies using universally accepted standards for communication and data should be enhanced and that identifying patients with high likelihood for success would be helpful to all parties involved. Future collaborative communications might include, but not limited to, monitoring patients via telemedicine and employing wearable technology, particularly cloud-based systems that can be accessed by the diverse healthcare team.

### 2.3 Expanded scope for monitoring is needed for serial assessments and therapy titration

Most diagnostic approaches currently provide one night of data. Many factors can contribute to night-to-night variability including patient familiarity with the equipment, changes in body posture, sleep stage distribution, alcohol intake, nasal congestion, etc. Multiple nights of data would be helpful in solidifying or informing context toward diagnosis (Stoberl et al., 2017). In addition, over time many factors can change including body weight or during titration of therapy, such that serial data over weeks or months would be helpful in guiding treatment for some patients.

A major contributor to optimizing therapy adherence is patient engagement which can be enhanced by providing objective data (Malhotra et al., 2018), e.g., improvement in OSA and metabolic risk in the context of weight loss (Chirinos et al., 2014). In the case of oral appliances, tracking residual OSA while using treatment can be helpful since additional mandibular advancement and/or other adjustments could be made, guided by objective data (Cistulli and Gotsopoulos, 2004). In patients with substantial comorbidities, e.g., COPD or CHF, serial data would be helpful in guiding sleep therapy particularly given the dynamic nature of these diseases. In some cases, exacerbations of COPD or CHF may be predicted by deterioration in sleep parameters as a harbinger of impending decompensation (Shorofsky et al., 2019; Do et al., 2022a,b). Early identification of patients at risk of hospitalization or readmission may allow targeted interventions potentially resulting in reduced healthcare costs (Light et al., 2018; Sterling et al., 2022).

### 2.4 Provider shortage and burnout accelerate need for updated care models

As previously stated, given the global burden of disease, the number of board-certified sleep specialists is unlikely adequate to address the volume of patients affected and at risk. Even among sleep technologists, respiratory therapists and nurses, there are current shortages in adequately trained personnel as many are choosing alternative job opportunities. Existing providers and staff are experiencing burn out due to multiple complex factors. However, the addition of extra data may impose a further burden on some individuals. For example, endocrinologists experienced a considerable workload with the advent of continuous glucose monitoring since the volume of data markedly increased, placing a burden on already stretched providers (Rodbard, 2016; Verbraecken, 2021).

Automation may be one solution to simplify routine tasks since a sophisticated algorithm could provide robust summary data and perhaps flag any outliers requiring expert review. Patient engagement is another strategy whereby the extra data become the responsibility of the patient (with appropriate disclaimers) and can be used to create peer group motivation rather than an extra burden on providers (Hoy et al., 1999). Nonetheless, expert providers will clearly be required on an ongoing basis for review of outlier data, for quality control performing routine audits of a percentage of patients to provide reassurance, and for concierge patients who prefer traditional models of care. Members of the sleep team like psychologists, nurse practitioners, physician assistants and sleep navigators with specialized and focused training are also a resource for consideration.

### 2.5 Financial models are needed that reward high quality care

Today, many US health systems use volume-based purchasing rather than value-based purchasing. The volume approach rewards *quantity* of care whereas in theory the value-based system rewards *quality* of care. Clearly, both approaches have merit since a variety of financial models are needed as the cost to provide care increases and budgets become more constrained. In a volume-based scenario, the development of care management service codes by US Medicare could be used to reward efforts used for remote patient or physiological monitoring (RPM) and may be one tool to improve outcomes and generate revenue. In addition, if increasing automation and assistant scoring of sleep studies occurs, the loss of professional revenue from interpreting reports could be addressed by rewarding high quality care based on avoiding subsequent health care costs (Pittman et al., 2004; Malhotra et al., 2013). However, we anticipate with improved efficiencies, increased volume would preserve financial viability.

In a value-based scenario, careful consideration must be given to the selection of robust validated quality metrics. Objectively captured data could be used to identify and define leading indicator(s) that predict important patient centric outcomes which are often lagging indicators. Some recent data suggest physician time expended on trying to achieve certain quality metrics does not yield better patient outcomes (Panzer et al., 2013; Saver et al., 2015; Adler, 2018). It is possible that OSA may be a disease that that could be part of a well-designed value-based care model, as evidence grows about the associated benefits of OSA treatment with cardiovascular risk reduction, reduction of hypertension, improvement of neurocognitive outcomes, etc. Additionally, evidence is growing with respect to the association of OSA treatment and reductions in health care resource use and costs. For example, treating OSA may reduce costs of various other interventions by preventing medical complications, motor vehicle accidents, etc. (Ayas et al., 2006; Strohl et al., 2013). In the case of chronic obstructive pulmonary disease, recent data showed a reduction in ER visits and hospitalizations with the consistent use of PAP therapy for OSA as compared to patients not consistently using PAP (Sterling et al., 2022).

## 3 Future directions and models

### 3.1 Establish a centralized specialist model

One solution that has been proposed for highly prevalent diseases is to develop specialized expertise for the care of these patients. In Africa, both HIV and tuberculosis are highly prevalent, and the volume of patients can easily overwhelm the existing infrastructure. Some providers have been specifically trained for the care and management of these patients to allow highly efficient/high-quality care. This approach allows up to 20 medications to be prescribed at one time with the experience of the provider enabling rigorous management of medication side-effects, drug interactions and identification of outliers that may require sub-specialty care. In the case of OSA, in theory one provider with multispecialty training could manage obesity, diabetes, hypertension and OSA rather than multiple individual sub-specialists. Recent data suggest weight loss through diet and exercise in conjunction with PAP therapy may be an effective treatment strategy for cardiometabolic risk in OSA (Chirinos et al., 2014; Carneiro-Barrera et al., 2022). Thus, OSA therapy including risk factor modification may yield superb outcomes. Clearly, these specialized providers would need to work within the context of a supportive environment including excellent communication with sub-specialists (Kalonji and Mahomed, 2019).

### 3.2 Triage to fast-track straight-forward cases model

For patients with high pre-test probability, an expedited pathway could be designed whereby patients have a telemedicine evaluation to initiate care management. Diagnostic testing and treatment could then be facilitated by telemedicine for straight-forward cases. This model can also evolve over time with a focus on continually simplifying the patient journey to access care. Adaptation during the crisis of the recent pandemic have helped to realize what could be the model for the future of clinical sleep medicine (Malhotra and Ayas, 2020). Likewise, more difficult cases would be scheduled with the appropriate specialist consult to coordinate more involved care management.

### 3.3 Tiered-care model

Given that management of some OSA patients is straight-forward, many patients do not require a face-to-face meeting with a physician specialist. A percentage of patients could likely be managed by primary care nurse practitioners, physician assistants, respiratory therapists, or sleep technologists (under a physician's supervision) though the more complicated cases may need direct physician evaluation, but only a subset would require seeing a sleep expert. Such an approach would need to be studied rigorously to ensure optimum care is provided. Clearly, communication would need to be robust between all tiers of care providers. Sleep navigators have been used effectively to triage patients within referring practices and to improve end-to-end patient care.

### 3.4 Predictive model(s)

In theory, predictive models and patient preferences could drive treatment decisions regarding who should receive oral appliances, upper airway surgery, or PAP therapy rather than based on “first come-first serve” approaches.

## 4 Discussion

Problems and unmet needs persist regarding the management of OSA following a consensus conference of key stakeholders to discuss potential future approaches to improve sleep apnea care and management. Other stakeholders will expand the scope of their solutions to close the gaps between problems, unmet needs, and available solutions. This scenario results in riding the bus driven by others. Or we can drive the bus to have more control over possible outcomes.

We should aim to simplify the patient's journey to achieve successful diagnosis and treatment. Delete what isn't required. Barriers to effective communication between providers and between providers and patients should be eliminated. Serial monitoring for OSA should be routine to establish reliable baseline measurements or monitor response to therapy over weeks and months. Provider shortages and burnout persist. These pose challenges when the demand for services increases, but the supply cannot. And most financial models still reward the quantity of care vs. the quality of care.

Solutions are possible when the entire ecosystem is engaged that manages and delivers care to patients. Communication between a sleep specialist and a sleep dentist to provide effective oral appliance therapy to a patient should not be limited to fax machines just because medical and dental information systems are not interoperable. We need to find solutions. This allows data regarding patient progress to also flow from the sleep dentist back to the sleep specialist.

Future directions and models should deliver more value to patients and improve the quality of their care. In value-based models, these are the core outcomes on which payment to treat patients is based. The efficiency in which a care team provides this care is also important to help manage resources. The entire ecosystem will need to be engaged to design, test, measure and iterate the technology, processes and care delivered through these models. Patients should win if we are successful.

## Data availability statement

The original contributions presented in the study are included in the article/supplementary material, further inquiries can be directed to the corresponding author.

## Author contributions

SP: Conceptualization, Project administration, Supervision, Writing – original draft, Writing – review & editing. BC: Conceptualization, Writing – original draft, Writing – review & editing. DG: Writing – review & editing. DH: Writing – review & editing. DK: Writing – review & editing. NS: Writing – review & editing. KS: Writing – review & editing. KT: Writing – review & editing. TB: Writing – review & editing. JT: Writing – review & editing. KC: Writing – review & editing. RB: Writing – review & editing. AR: Writing – review & editing. SA-I: Writing – review & editing. AM: Conceptualization, Writing – original draft, Writing – review & editing.
